# Bead–Spring Simulation of Ionomer Melts—Studying the Effects of Chain-Length and Associating Group Fraction on Equilibrium Structure and Extensional Flow Behavior

**DOI:** 10.3390/polym15234560

**Published:** 2023-11-28

**Authors:** Supun S. Mohottalalage, Andrew P. Saab, Amitesh Maiti

**Affiliations:** Lawrence Livermore National Laboratory, Livermore, CA 94550, USA; kamkanammoho1@llnl.gov (S.S.M.);

**Keywords:** associative polymers, bead–spring model, molecular dynamics, extensional rheology

## Abstract

Ionomers are associative polymers with diverse applications ranging from selective membranes and high-performance adhesives to abrasion- and chemical-resistant coatings, insulation layers, vacuum packaging, and foamed sheets. Within equilibrium melt, the ionic or associating groups are known to form thermally reversible, associative clusters whose presence can significantly affect the system’s mechanical, viscoelastic, and transport properties. It is, thus, of great interest to understand how to control such clusters’ size distribution, shape, and stability through the designed choice of polymer architecture and the ionic groups’ fraction, arrangement, and interaction strength. In this work, we represent linear associating polymers using a Kremer–Grest type bead–spring model and perform large-scale MD simulations to explore the effect of polymer chain-length (l) and fraction ($f_{s}$) of randomly placed associating groups on the size distribution and stability of formed clusters. We consider different chain-lengths (below and above entanglement), varying fractions of associating groups (represented by ‘sticky’ beads) between 5 and 20%, and a fixed sticky–sticky nonbond interaction strength of four times that between regular non-associating beads. For all melts containing associating groups the equilibrium structure factor S(q) displays a signature ionomer peak at low wave vector q whose intensity increases with increasing $f_{s}$ and l. The average cluster size $N_{c}$ increases with $f_{s}$. However, the effect of chain-length on $N_{c}$ appears to be pronounced only at higher values of $f_{s}$. Under extensional flows, the computed stress (and viscosity) is higher at higher $f_{s}$ and l regardless of strain rate. Beyond a critical strain rate, we observe fragmentation of the associative clusters, which has interesting effects on the stress/viscous response.

## 1. Introduction

Associating polymers are smart, responsive materials that incorporate reversibly associative functional groups. They have been used in diverse technologies ranging from water purification [1,2] to desalination [3,4], fuel cells [5,6], biomedical sensors [7,8], flow batteries [9,10], enhanced oil recovery in petroleum industry [11,12], and self-healing [13,14] applications. Ionomers are an important subclass of associating polymers in which less than 20% of the backbone atoms are bonded to pendant groups that are ionic, with nonbonded counterions maintaining charge neutrality. The associating groups in ionomers tend to form aggregates (also referred to as *clusters*) [15] within the bulk microstructure, as have been captured by previous theoretical [16,17,18], experimental [19,20,21,22,23,24], and simulation [25,26,27,28,29] studies. One of the first theoretical models of cluster formation in ionomer melts was proposed decades ago by Eisenberg [16,17]. In this model, the associating groups first form multiplets, which grow into clusters. The sizes, shapes, and stability of the clusters are governed by factors such as backbone flexibility and the fraction and binding strength of the associating groups [16]. Experimental studies by Winey et al. [19] later detected spherical aggregates in small-angle X-ray scattering (SAXS) and scanning transmission electron microscope (STEM) images. Recent simulation studies [26,27,28,29] have also captured the formation of clusters, and the corresponding computed static structure factor S(q) was in good agreement with available experimental results.

Besides being structurally interesting, the formation of clusters can lead to significant changes to the physical, mechanical, and viscoelastic properties of an ionomer melt. Over the past two decades, several studies [21,22,23,26,29,30,31,32,33] have captured the effects of associating groups on the polymer structure, dynamics, and rheology. For sulfonated polystyrene (SPS) systems, SAXS measurements revealed a peak under light sulfonation levels [21], indicative of the formation of clusters. As the sulfonation fraction was increased, the feature developed into a sharp, intense peak. More recently, there have been a number of computational explorations of cluster formation in associating polymer melts using both coarse-grained [31] and fully atomistic [25,26,29] simulations. In particular, Senanayake et al. [31] investigated the structure and dynamics of melts of fully flexible, linear associating polymers using the Kremer–Grest bead–spring model in which the interaction strength between associating beads was varied from 1 to 20$\;k_{B}T$. It was found that with an increase in association strength the average cluster size increases, which is accompanied by a dramatic decrease in the mobility of the polymer chains. Fully atomistic molecular dynamics have also been used to explore associating polymer melts. For instance, Agrawal et al. [25] and Mohottalalage et al. [26] investigated the structure factor S(q) of SPS melts below the entanglement length and found excellent agreement with available experimental data. Mohottalalage et al. [26] further demonstrated that parameters such as the size of the clusters and the number of unique polymer chains associated with clusters can have a considerable impact on the local dynamics of the polymer. 

Understanding the rheological properties of associating polymers is also of great interest, given that these materials are hard to formulate and process. Weiss and coworkers performed a few of the first rheological experiments in this field [22,32,33,34,35]. They observed that a small number of ionic groups can significantly impact the viscoelastic behavior of SPS. Ling et al. [35] studied the extensional behavior of SPS melts (~6.5 mol % sulfonation) and observed pronounced extensional stress effects for ionomers at higher strain rates and lower temperatures. Further investigations revealed that the strain at the stress maximum for ionomers is substantially lower than that in non-sulfonated polystyrene (PS) melts. They speculated that the stress maximum could be due to disruptions of the ionomer-cluster network [35]. Yu et al. [33] studied the linear viscoelastic behavior of lightly (~0.11–1.71 mol %) sulfonated SPS ionomers. They reported that the rubbery modulus increased with an increasing sulfonation percentage and followed the classical rubber elasticity theory. A recent rheology study by Mohottalalage et al. [30] for coarse-grained melts of fully flexible associating polymers showed that while the equilibrium cluster size expectedly increases with an increase in association strength, extensional flow leads to a dynamic equilibrium in which the clusters break and reform continuously. This study was recently extended to SPS systems using all-atomistic simulations [36].

The present work aims at exploring a few aspects of ionomer systems that have not been systematically studied before, i.e., the effect of the chain-length (below and above entanglement length) and the fraction of associating groups on the equilibrium-structure and the viscoelastic properties of associating polymer melts under extensional flow. Given significant computational requirements, we have employed bead–spring-level simulations here, with the possibility of all-atom modeling of technologically important ionomer materials in our future efforts.

## 2. Methodology

We performed equilibrium and non-equilibrium MD simulations using the Large-scale Atomic/Molecular Massively Parallel Simulator (LAMMPS) [37] and the standard Kremer–Grest bead–spring model [38,39], which describes the fully flexible linear polymer chains. In this model, each polymer chain is represented by a string of beads, each bead physically representing a Kuhn monomer. Nearby beads (with 1–2, 1–3, and 1–4 exclusion for neighboring intra-chain beads) interact via a pairwise nonbond Lennard-Jones (LJ) potential within a cutoff range of 2.5σ, while neighboring beads on a chain are connected by finitely extensible nonlinear elastic (FENE) springs with a mean bond length of *b* ≈ 0.96σ. Two types of beads are employed, i.e., ‘regular beads’ representing non-associating groups α and ‘sticky beads’ representing associating groups β. While the FENE spring potential and the mass are the same for all beads, the LJ potential interaction is stronger for the sticky beads. In this work, we made the choice $\varepsilon_{\beta \beta }=4\varepsilon_{\alpha \alpha }$ with the cross-term $\varepsilon_{\alpha \beta }=\varepsilon_{\alpha \alpha }$. Such interactions are typical of ionomer association strengths that are known to yield cluster formation [30,31]. In the discussion below, we express each quantity in *reduced units*, i.e., mass is expressed in units of mass per bead m, length is expressed in units of LJ parameter σ, energy is expressed in units of LJ parameter $\varepsilon_{\alpha \alpha }$, temperature is expressed in units of $k_{B}T/\varepsilon_{\alpha \alpha }$, and time is expressed in units of $\tau =\sigma \sqrt{m/\varepsilon_{\alpha \alpha }}$.

We studied melts equilibrated at temperature *T* = 1 and density *ρ* ≈ 0.87, using a time step Δ*t* = 0.001. Each melt was represented by 500 chains of equal length. Four different chain-lengths were explored, i.e., l = 20, 40, 80, and 120 beads. To understand the effect of association group fraction on the size and stability of formed clusters, we considered three different sticky fractions, i.e., $f_{s}\;$= 5, 10, and 20%, with sticky beads placed randomly along the chains.

For each system, the initial structures were created using the ‘standard’ procedure described in section V of Auhl et al. [40]. Briefly, the procedure consists of four steps: (1) Start from an ensemble of chains with the correct end-to-end distance, i.e., $R^{2}(N)\propto (N-1)$. (2) Place the chains randomly in a large simulation box (i.e., at much lower density than the equilibrium bulk density). (3) Introduce a soft excluded-volume potential between non-connected beads, which is of the functional form $A\left(1+\cos\left(\pi r/r_{c}\right)\right)$ (for $r\leq r_{c}=2^{1/6}\sigma$) with an initial amplitude $A=4\varepsilon_{\alpha \alpha }$, which is linearly increased to a final value of $A\geq 100\varepsilon_{\alpha \alpha }$ (‘push-off’) over a short time interval (10−20 τ). This procedure leads to an elimination of all initial atom-overlaps and results in large enough inter-monomer distances that enables using the LJ potential without any instabilities. (4) Relax the system with the full LJ potential with a short MD run. The ‘standard’ procedure described above is sufficient for chains of length up to 120 as studied in the present work. For much longer chains (500 or above), a more sophisticated procedure could become necessary [40]. Each initial structure was subjected to an equilibration run of length $0.4\times 10^{6}\tau$ (i.e., $0.4\times 10^{9}$ time steps). To ensure that a state of equilibrium had been reached, we monitored the time-evolution of a few structural characteristics, including the average chain end-to-end distance, radius of gyration, and radial distribution function. Following equilibration, a production NVT run of length $10^{6}\tau$ was carried out for each system.

Finally, extensional flow simulations on the melt systems were performed by employing the Generalized Kraynik–Reinelt (GKR) [41,42] boundary conditions under a constant Hencky strain rate $\dot{\varepsilon }\equiv \partial \ln\Lambda /\partial t$, where Λ is the stretch ratio along the z direction. The GKR boundary condition enables the achievement of much larger strains without collapsing the simulation box in the contracting directions [30,41,42,43]. The equations of motion under flow were integrated using the g-SLLOD algorithm [44,45] as implemented in LAMMPS. We computed quantities such as extensional stress $\sigma_{E}=\sigma_{zz}-\left(\sigma_{xx}+\sigma_{yy}\right)/2$ and the transient extensional viscosity $\eta_{ex}(\varepsilon )=\sigma_{E}/\dot{\varepsilon }$. To correlate with experimental results [46,47], we expressed the applied strain rate in terms of a dimensionless Weissenberg number [48,49,50] defined as $Wi=\dot{\varepsilon }\tau_{R}$, where $\tau_{R}$ is the Rouse time, given by $\tau_{R}=\tau_{e}Z^{2}$, where $Z=l/l_{e}$ is the average number of entanglements per chain and $\tau_{e}$ the entanglement time. For linear and fully flexible bead–spring polymers, $l_{e}\sim 84$ [51,52] and $\tau_{e}\sim 10^{4}\tau$ [53], which enables relating Wi to applied strain rates $\dot{\varepsilon }$. In this work we vary Wi from 4 to 32. From the above value of $l_{e}$ we also note that our chosen chain-lengths of l=20, 40, 80, 120 cover cases of both below and above entanglement length.

## 3. Results and Discussion

### 3.1. Equilibrium Study

**Figure 1:**
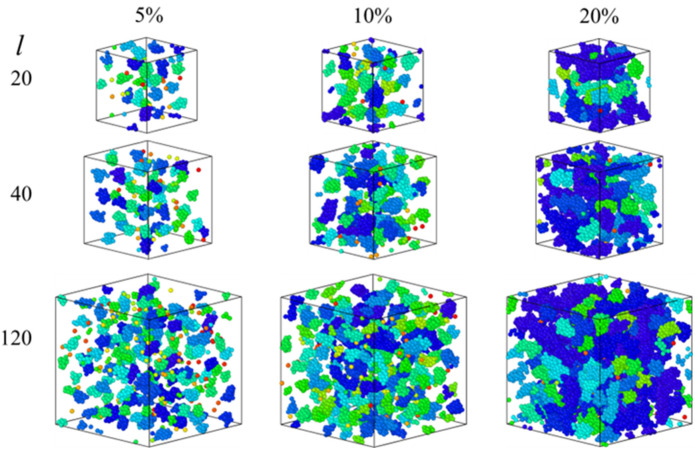
Snapshots of associated (sticky) clusters in melts for chain-lengths: l=20, 40, and 120 for sticky fractions; $f_{s}=$5% (**left**), 10% (**middle**), and 20% (**right**). Red, green, blue indicates isolated, medium-sized, and large clusters.

Figure 1 illustrates snapshots of clusters formed in melts of chains with varying lengths and sticky fractions. We define a cluster as a collection of sticky beads such that each sticky bead has at least one sticky bead neighbor within a distance of 1.5σ. The clusters in Figure 1 are colored based on their size (i.e., the number of sticky beads present within the cluster) using a red–green–blue color scheme, with single sticky beads in red and medium-sized and larger clusters in green and blue, respectively. Even at $f_{s}=$ 5% significant clustering effects are observed (Figure 1 (left)). Such results are similar to Kirkmeyer et al. [19] who observed ionic aggregates in ionomers using STEM imaging. Figure 1 also shows that the average cluster size increases with increase in $f_{s}$, while the effect of chain-length on overall cluster sizes appears weak. The formation of a typical cluster about average size in a polymer melt corresponding to l=120 and $f_{s}=$ 5%. is shown in Supplementary Section (Video S2).

**Figure 2:**
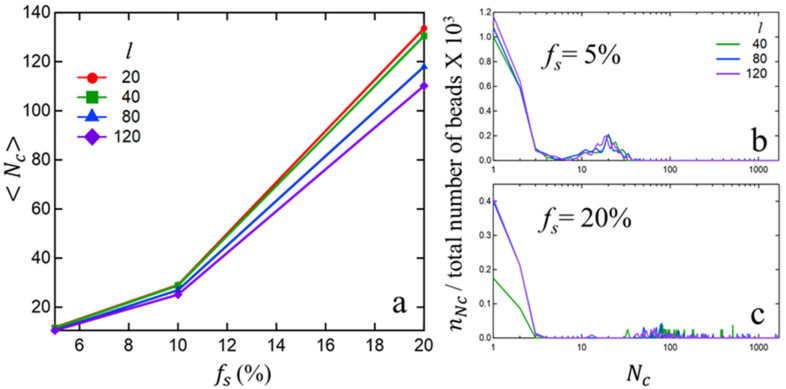
(**a**) Average cluster size $\langle N_{c}\rangle$ vs. sticky fraction $f_{s}$ (%), for different chain-lengths l. (**b**,**c**) size distribution of clusters $n_{Nc}$ (normalized by the total number of beads) as a function of cluster size $N_{c}$ for two different sticky fractions $f_{s}$.

To more precisely quantify the effect of chain-length l and sticky fraction $f_{s}$ on cluster sizes, we have computed the cluster size distribution within each melt. Figure 2a plots the average cluster size $\langle N_{c}\rangle$ as a function of the sticky fraction $f_{s}$, while Figure 2b plots the size distribution of clusters $n_{Nc}$ (normalized by the total number of beads in the simulation cell) for $f_{s}=5\%$ and 20%. 

It is interesting to note that, at a low sticky fraction ($f_{s}=5\%$), the average cluster size (and the cluster size distribution on whole) is insensitive to the polymer chain-length l. However, at higher sticky fractions, the average cluster-size decreases monotonically with chain-length, with a sharper decrease around and above the entanglement length. Figure 2c provides deeper insight into this phenomenon by showing that the number of ‘isolated sticky beads’ ($N_{c}=1$) is significantly higher for l=120 as compared to l=40. One can attribute such behavior to entanglement-induced constraints to structural relaxation of larger chains as compared to shorter chains, which hinders the aggregation process of sticky beads into larger clusters. Additionally, when comparing Figure 2b,c, one observes a much wider distribution of cluster sizes at a larger sticky fraction (for chains of all sizes), a clear indication of a combinatorically much larger number of choices of cluster formation in the presence of a larger number of associating groups.

**Figure 3:**
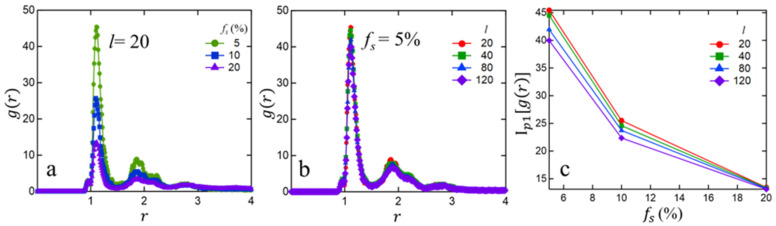
Sticky bead–sticky bead radial distribution function g(r), for (**a**) a fixed chain-length (l=20) and different sticky fractions ($f_{s}$) and (**b**) for a fixed $f_{s}$ (5%) and different l. (**c**) Intensity of the first peak $I_{p1}[g\left(r\right)]$ as a function of $f_{s}$ for different chain-lengths.

To better understand the compactness of the clusters, we computed the sticky bead–sticky bead radial distribution function g(r) (normalized by the total number of sticky beads in the system) as plotted in Figure 3. The first peak, at approximately 1.1σ, corresponds to the shortest distance between two associating beads within a cluster. The intensity of the first peak ($I_{p1}[g\left(r\right)]$) is significantly higher than the second and third peaks, which are at approximately 1.9σ and 2.8σ, respectively. The second and the third peaks are broader than the first and correspond to the next neighboring sticky beads within the cluster. From Figure 3, we observe that for smaller $f_{s}$ the intensity of the first peak is higher, which indicates a more compact (i.e., spherical-like) clusters for smaller sticky fractions. At higher $f_{s}$ the clusters are bigger (see Figure 1 and Figure 2) but less compact, i.e., more ‘irregular’ and/or ‘extended’ shapes. With increasing chain-length l, the cluster compactness modestly decreases at small sticky fractions, likely reflecting higher relaxation freedom for smaller chains. However, at high $f_{s}$ (20%) the degree of cluster compactness deteriorates markedly for all chain-lengths, and the chain-length dependence of $I_{p1}[g\left(r\right)]$ becomes insignificant. A significant departure of cluster shapes from simple spherical has previously been observed in all-atomistic simulations [26,54].

**Figure 4:**
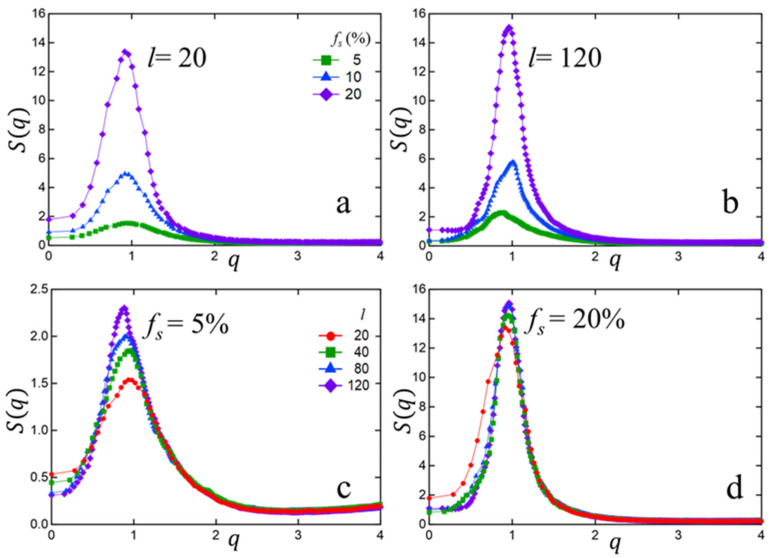
Structure factor $S\left(q\right)$ of sticky beads as a function of momentum transfer wave vector *q* for different chain-lengths *l* = 20 (**a**) and 120 (**b**) and sticky fractions $f_{s}$ = 5% (**c**) and 20% (**d**). We focus on the small-q region (<4 in units of 2π/σ), which displays a single peak representative of inter-cluster ordering.

To quantify the degree of spatial inter-cluster ordering of the associated clusters within the bulk polymer matrix, we computed the static structure factor of the sticky beads, defined as $S\left(q\right)=N^{-1}\langle {\left|\sum_{j=1}^{N}\exp\left(iq.r_{j}\right)\right|}^{2}\rangle$, where $r_{j}$ is the position vector of the $j^{th}$ sticky bead, N is the total number of sticky beads in the system, and $\langle \ldots \rangle$ indicates equilibrium averaging over MD trajectory frames. Results are summarized in Figure 4 for different chain-lengths and sticky fractions. We focus on the small-q region of four wave vectors (in units of 2π/σ) or fewer, which contains a single prominent peak characteristic of inter-cluster correlation. Such an ionomer peak directly correlates with the average Inter-cluster distance and has been previously observed in MD simulations [26,29,54,55] and small-angle X-ray scattering (SAXS) [19,56,57]. Figure 4 displays several interesting results, including the following: (1) For a fixed chain-length, the peak intensity significantly increases with an increase in sticky fraction $f_{s}$, with little change in peak position. This is likely due to larger cluster sizes at higher sticky fractions while inter-cluster distances remain relatively unchanged. (2) At low sticky fraction ($f_{s}=5\%$), the peak position shifts slightly leftward with an increase in chain-length, while the peak intensity increases. This indicates that, for longer chains, the clusters are distributed with a somewhat higher degree of spatial uniformity and spaced out slightly further apart. And (3) at higher sticky fraction ($f_{s}=20\%$) the dependence of S(q) on chain-length becomes much smaller, likely due to complex effects resulting from a much wider dispersion in cluster sizes.

**Figure 5:**
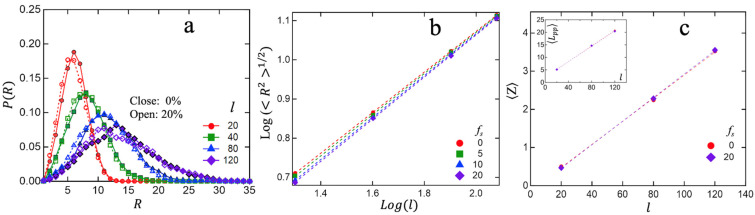
Comparing equilibrium structural properties of unassociated ($f_{s}=0\%;close-solid\;symbols$) and associated ($f_{s}=20\%;open-dash\;symbols$) melts: (**a**) Distribution function $P\left(R\right)$ of the intra-chain end-to-end distance R; (**b**) rms end-to-end distance ${\langle R^{2}\rangle }^{1/2}$ as a function of chain-length (l) for different $f_{s}$; (**c**) mean number of kinks per chain $\left\langle Z\right\rangle$, with the corresponding mean contour length of the primitive paths $\left\langle L_{pp}\right\rangle$ shown as inset.

While the formation of associative clusters is a well-established phenomenon for ionomers, there has been some controversy in the literature about how such clusters affect the equilibrium structural properties of the polymer melt. For instance, certain theories [18,58] predict significant chain expansion in ionomers, while a different theoretical study [59] and a neutron scattering study [60] conclude no apparent chain expansion due to clustering. To shed light on this issue, we computed the equilibrium distribution of the intra-chain end-to-end distance $P\left(R\right)$. Figure 5 displays results for the unassociated system ($f_{s}=0\%$) and the system with $f_{s}=20\%$. We conclude that the formation of associated clusters has little effect on the overall structural properties of the chains in the melt. In particular, the distribution of end-to-end distance P(R) (Figure 5a) and the rms end-to-end distance ${\langle R^{2}\rangle }^{1/2}$ as a function of chain-length (Figure 5b) follow the behavior expected of Gaussian chains. To analyze the state of chain entanglement within the melts, we have also carried out primitive path (PP) analysis using the Z1+ code from Kröger et al. [61]. Figure 5c plots the average number of entanglements ($\langle Z\rangle$) per chain (defined in terms of topological ‘kinks’) and the average contour length of the primitive path ($\langle L_{pp}\rangle$) (Figure 5c inset) as a function of chain-length. We observe the expected linear behavior as a function of chain-length with no noticeable effect due to associative clusters. The average number of kinks per chain also appears consistent with an entanglement length $l_{e}\sim 84$, as has been known from previous analysis of such bead–spring melts [51,52].

### 3.2. Ionomers under Extensional Flow

**Figure 6:**
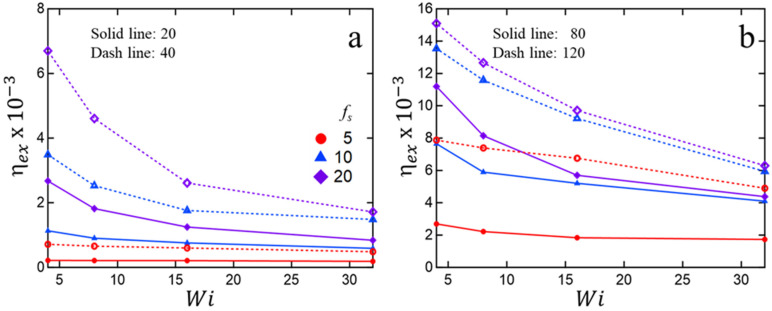
Terminal extensional viscosity $\eta_{ex}$ as a function of Weissenberg number Wi for different chain-lengths (l) and sticky fractions ($f_{s})$. For clarity, four different chain-lengths are split into two figures: (**a**) l=20, 40; (**b**) l=80, 120.

**Figure 7:**
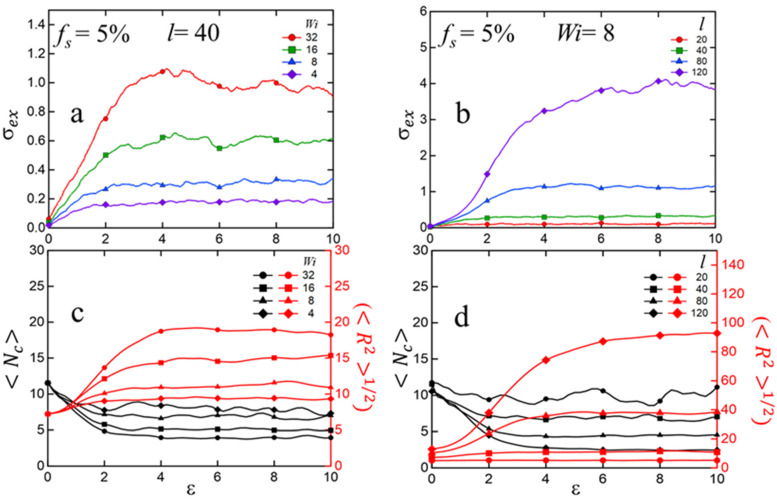
Extensional stress ($\sigma_{ex}$), average cluster size ($\langle N_{c}\rangle$), and rms intra-chain end-to-end distance (${\langle R^{2}\rangle }^{1/2}$) as a function of Hencky strain (ε) for melts with a fixed sticky fraction $f_{s}=5\%$: (**a**) $\sigma_{ex}$ for l=40 and different Wi; (**b**) $\sigma_{ex}$ for Wi=8 and different l; (**c**) $\langle N_{c}\rangle$ and ${\langle R^{2}\rangle }^{1/2}$ for l=40 and different Wi; (**d**) $\langle N_{c}\rangle$ and ${\langle R^{2}\rangle }^{1/2}$ for Wi=8 and different l.

**Figure 8:**
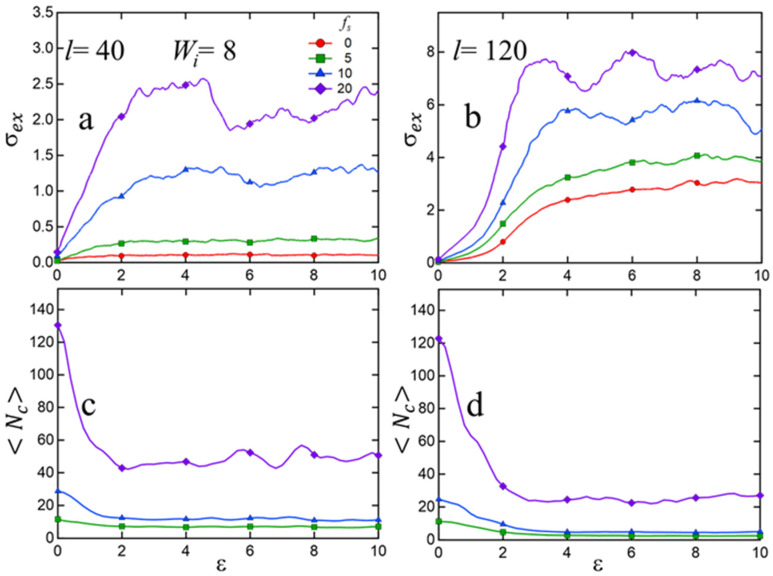
Extensional stress ($\sigma_{ex}$) and average cluster size ($\langle N_{c}\rangle$) as a function of Hencky strain (ε) for different sticky fractions at a fixed strain-rate (Wi=8) for two different chain-lengths (l): (**a**) $\sigma_{ex}$ for l=40; (**b**) $\sigma_{ex}$ for l=120; (**c**) $\langle N_{c}\rangle$ for l=40; (**d**) $\langle N_{c}\rangle$ for l=120.

The processing of ionomer melts under different flow conditions is of great technological interest. Several studies in the associating polymer literature have shown significant correlation between the strength of association and rheological properties, including the ease or difficulty of processability [62,63,64,65,66]. To this end, we simulated flow under a constant Hencky strain rate $\dot{\varepsilon }$ (or equivalently, constant Weissenberg number (Wi), see Section 2) for melts of different chain-lengths and sticky fractions. Figure 6 plots the terminal extensional viscosity ($\eta_{ex}=\sigma_{ex}/\dot{\varepsilon }$ for large ε, where $\sigma_{ex}$ is extensional stress as defined in Section 2) as a function of Wi. In all cases, we observe shear thinning behavior and an enhancement of viscosity with increasing chain-length and sticky fraction. To provide further insight, we have analyzed the extensional stress response and average cluster size in Figure 7 and Figure 8. 

Figure 7a,b show the stress–strain behavior of the $f_{s}=5\%$ ionomers for various strain rates (Wi) and chain-lengths (l), respectively. We see a linearly increasing extensional stress $\sigma_{ex}$ for (Hencky) strain up to ε~4. With further increase in strain, the stress $\sigma_{ex}$ fluctuates around a steady-state value. This steady-state value is used to compute the terminal viscosity $\eta_{ex}$ in Figure 6. From Figure 7a,b, we see that the steady-state $\sigma_{ex}$ increases with increasing strain-rate as well as increasing chain-length. Figure 7c,d display the corresponding mean cluster size $\langle N_{c}\rangle$ and rms end-to-end distance ${\langle R^{2}\rangle }^{1/2}$, which reveal the following: (1) the steady-state average cluster-size decreases both with increasing strain (i.e., flow) rate and with increasing chain-length (MD movie showing the extension of the polymer chain is presented in the Supplementary Section; Video S2), which leads to a larger number of clusters and consequently supports higher levels of extensional stress, and (2) at a given flow rate (Wi=8) longer chains stretch proportionally longer, as evident from the ratio ${\langle R^{2}\rangle }^{1/2}:l$. This phenomenon is likely due to a larger number of clusters in the steady state, which, on an average, leads to a larger tensile pull on the chains along the flow direction.

Figure 8 explores extensional stress and average cluster size at a fixed flow rate (Wi=8) for various sticky fractions $f_{s}$ and two different chain-lengths. We observe that both extensional stress and average cluster-size in steady-state flow increase with increasing sticky fraction $f_{s}$. Additionally, we also see that at higher sticky fractions (10% and higher) a stress overshoot develops that is not observable at low $f_{s}$. Such behavior in strongly associating polymers has been documented in previous experimental [22,67,68] and simulation studies, both all-atom [36] and coarse-grained [30]. 

**Figure 9:**
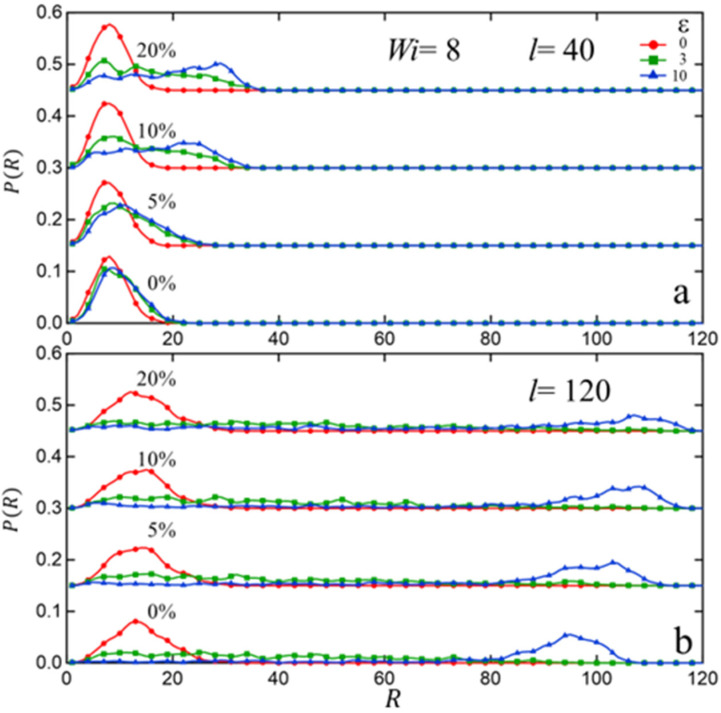
(**a**) Distribution function P(R) of the intra-chain end-to-end distance R for a fixed strain rate (Wi=8) and different sticky fractions ($f_{s}$) at three different values of Hencky strain (ε) and two different chain-lengths: (**a**) l=40; (**b**) l=120. For clarity, curves for $f_{s}=$5, 10, and 20% are vertically shifted by 0.15, 0.30, and 0.45, respectively.

To gain a deeper perspective on the effect of extensional flow on individual chains, we computed the distribution $P\left(R\right)$ of intra-chain end-to-end distance R. Figure 9 displays these results for systems with chain-lengths l=40 and l=120 for sticky fractions $f_{s}=0,\;5,\;10,\;20\%$ for the initially unstrained state (ε=0) and two different strain states (ε=3, 10). For the non-associated melt ($f_{s}=0\%$), we see two different behaviors depending on whether chains are unentangled (l=40) or above entanglement length (l=120), i.e., the unentangled chains do not stretch much under flow relative to its equilibrium, while most chains above entanglement length stretch under flow, as shown by the blue peak. On the other hand, for associated melts the formation of clusters acts as effective cross-links and leads to significant chain stretching (near full extension) with the effect being more pronounced at higher sticky fractions. In all cases, the P(R) distribution appears to reach a steady state profile, which is consistent with the behavior seen in Figure 7 and Figure 8 that display a steady state pattern for ε>4. 

**Figure 10:**
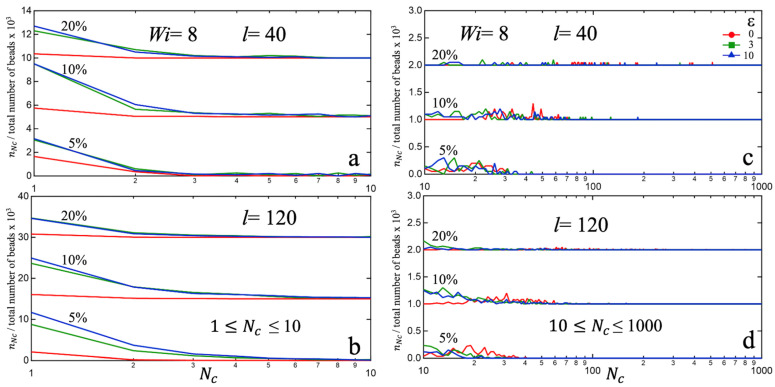
Cluster size distribution profiles for a fixed strain rate (Wi=8), two different chain-lengths (l=40, 80), three different sticky fractions ($f_{s}=5,\;10,\;20\%$), and three different strain levels (ε=0, 3, 10). For visual clarity, the curves for different sticky fraction $f_{s}$ are vertically shifted, and the cluster size-ranges are split into two intervals: (**a**,**b**) $1\leq N_{c}<10$ and (**c**,**d**) $N_{c}\geq 10$.

Figure 10 shows the cluster-size distributions corresponding to each $f_{s}>0$ figure in Figure 9. For a clearer display, the plots are split into two size ranges, i.e., $1\leq N_{c}<10$ and $N_{c}\geq 10$. In both ranges, we see a leftward shift of the size distribution $n_{Nc}$ with flow, which results from the flow-induced fragmentation of equilibrium clusters. It is interesting that the largest clusters in the unentangled melt (l=40) under equilibrium (ε=0) are larger than the largest clusters in the longer-chain melts, the size of the former being as large as a few hundred for $f_{s}=20\%$. Even after flow-induced fragmentation, a few large clusters can remain in the unentangled melt. This is likely due to the smaller unentangled chains have higher flexibility in aligning themselves in the flow direction. This leads to stress relaxation in the system and less cluster fragmentation as compared to longer-chain melts (l=120). A higher level of steady-state stress in associated longer-chain melts is subsequently maintained by the presence of a larger number of medium-sized fragmented clusters that act as effective cross-links.

## 4. Conclusions

In this study, bead–spring molecular dynamics simulations were employed to investigate the impact of chain-length (below and above entanglement length) and sticky fraction (up to 20%) on the structure and rheological behavior of associating polymer melts. Employing a sticky–sticky interaction strength of four times that between non-associating groups, we observed the formation of stable clusters at a sticky fraction $f_{s}$ of just 5%. With increasing $f_{s}$ the average cluster-size increases, while the cluster size distribution becomes wider. We also found that at low $f_{s}$ the average cluster-size and the size distribution are relatively insensitive to chain length l, while at higher $f_{s}$ smaller l leads to more efficient (and therefore larger) clusters. To characterize the compactness of clusters we computed the sticky–sticky radial distribution function g(r) in equilibrium melts and found that more compact clusters form at lower $f_{s}$, while at higher $f_{s}$ the clusters are bigger but less compact, i.e., more irregular and/or extended shapes. With increasing chain-length l the cluster compactness modestly decreases at small sticky fractions, likely due to more restricted chain relaxation. However, at high $f_{s}$ (20%) the degree of cluster compactness deteriorates irrespective of chain-length. To quantify the uniformity and separation in the spatial distribution of clusters, we computed the static structure factor S(q) and found that at lower $f_{s}$ (5%) the clusters in longer-chain melts are distributed with a somewhat higher degree of spatial uniformity and spaced out slightly further apart as compared to in unentangled melts. However, at higher $f_{s}$ such dependence on chain-length becomes less distinguishable due to complex effects of a much wider dispersion in cluster-sizes. To characterize whether cluster formation has any effects on the global structural properties of the individual chains, we computed the distribution P(R) of the intra-chain end-to-end distance R and found that clusters have little effect on such distribution. This likely implies that chain relaxations involved in cluster formation are ‘local’ in nature and do not involve large-scale rearrangements relative to unassociated melts.

Finally, under extensional flows all associated systems exhibited shear-thinning with a steady-state behavior for (Hencky) strain levels ε>4. The corresponding viscosity was found to be higher for larger sticky-fraction $f_{s}$ and longer chain-lengths l, as expected. Higher strain-rates were found to result in greater fragmentation of equilibrium clusters, which in dynamical equilibrium led to a decrease in average cluster-size. In unentangled melts (small chain-lengths) a higher level of stress relaxation occurs through increased flexibility of individual chains in aligning parallel to the flow direction. This leads to less cluster fragmentation and, thus, fewer but larger clusters. In contrast, for longer-chain melts under extensional flow a larger number of medium-sized fragmented clusters form, which act as effective cross-links. This, plus an additional effect of topological constraints due to entanglements, supports a higher level of extensional stress in longer-chain melts. Results on equilibrium and flow properties such as these can provide useful insights on how to control and optimize the processing of associated polymer melts for various applications. 

## Supplementary Materials

The following supporting information can be downloaded at https://www.mdpi.com/article/10.3390/polym15234560/s1, Video S1: Movie illustrating the formation of three average-sized random clusters in a polymer melt of chain-lengths l=120 and associating (i.e., sticky) bead fraction $f_{s}=5\%$. For clarity of visualization, only the sticky beads are displayed. The movie corresponds to a MD simulation run of $0.35\times 10^{6}\tau \;$(i.e., $0.35\times 10^{9}$ time-steps); Video S2: Movie showing the extension of polymer chains in a melt with l=120 and $f_{s}=20\%$, under extensional strain-rate Wi = 32, for true (Hencky) strain levels up to ε=10. Different colors have been employed to distinguish different polymer chains.
