# Supplementary material for: Bead–Spring Simulation of Ionomer Melts—Studying the Effects of Chain-Length and Associating Group Fraction on Equilibrium Structure and Extensional Flow Behavior
Source: Polymers (Basel). 2023 Nov 28;15(23):4560. doi: 10.3390/polym15234560 (PMC10708384; doi:10.3390/polym15234560)
Supplement: Supplementary file 1 [file polymers-15-04560-s001.zip › SI_Polymers_2697762.pdf]

Supplemental Information

Bead-spring Simulation of Ionomer Melts – Studying the Effects of Chain-length and Associating Group fraction on Equilibrium Structure and Extensional Flow Behavior

By Mohottalalage, Saab, and Maiti

120mers_f_5_cluster TRG.mp4

Video S1. Movie illustrating the formation of three average-sized random clusters in a polymer melt of chain-lengths $l = 120$ and associating (i.e., sticky) bead fraction $f_s = 5\%$. For clarity of visualization, only the sticky beads are displayed. The movie corresponds to a MD simulation run of $0.35 \times 10^6 \tau$ (i.e., 0.35×10^9 time-steps).

f20_120mers_wi32.mp4

Video S2. Movie showing the extension of polymer chains in a melt with $l = 120$ and $f_s = 20\%$, under extensional strain-rate Wi = 32, for true (Hencky) strain levels up to $\varepsilon = 10$. Different colors have been employed to distinguish different polymer chains.
